# Comparison of Two DNA Extraction Methods and Two PCRs for Detection of *Echinococcus multilocularis* in the Stool Samples of Naturally Infected Red Foxes

**DOI:** 10.3390/ani10122381

**Published:** 2020-12-11

**Authors:** Katarzyna Skrzypek, Jacek Karamon, Małgorzata Samorek-Pieróg, Joanna Dąbrowska, Maciej Kochanowski, Jacek Sroka, Ewa Bilska-Zając, Tomasz Cencek

**Affiliations:** Department of Parasitology and Invasive Diseases, National Veterinary Research Institute, Partyzantów Avenue 57, 24-100 Puławy, Poland; katarzyna.skrzypek@piwet.pulawy.pl (K.S.); malgorzata.samorek-pierog@piwet.pulawy.pl (M.S.-P.); Joanna.dabrowska@piwet.pulawy.pl (J.D.); maciej.kochanowski@piwet.pulawy.pl (M.K.); jacek.sroka@piwet.pulawy.pl (J.S.); ewa.bilska@piwet.pulawy.pl (E.B.-Z.); tcencek@piwet.pulawy.pl (T.C.)

**Keywords:** *Echinococcus multilocularis*, PCR, DNA extraction, feces

## Abstract

**Simple Summary:**

The goal of the study was to compare the efficiency of two commercial DNA extraction kits together with two different Polymerase Chain Reaction (PCR) protocols in the detection of *Echinococcus multilocularis* in the feces of naturally infected foxes. Stool samples from red foxes were collected in a highly endemic area in Poland. Sedimentation and counting technique (SCT) was used as a reference method. From 48 samples, 35 were positive in SCT. Further investigations showed that 40.0% of samples (from those with SCT positive result) after Z—DNA extraction and 45.7% after Q—DNA extraction gave positive results in nested PCR. In multiplex PCR, positive results were obtained in 54.3% of samples after Z isolation and 48.6% of samples after Q. Additionally, one sample negative in SCT gave a positive result in PCR. The number of worms detected in the intestines had no influence on the PCR results. Both of the extraction methods showed similar efficiency in DNA isolation and dealing with inhibitors; however, they showed relatively low sensitivity.

**Abstract:**

(1) Background: Due to the increasing distribution of *Echinococcus multilocularis* infections in final hosts, epidemiological investigations are important for recognizing the spreading pattern of this parasite and also to estimate risk infection for humans. (2) Methods: Investigations were conducted with two commercial kits dedicated for DNA extraction from feces: ZR Fecal DNA Mini Prep (Zymo Research, Freiburg, Germany) and QIAamp FAST DNA Stool Mini Kit (Qiagen, Hilden, Germany) (marked as Z and Q), together with two common PCR protocols (nested PCR and multiplex PCR). The goal was to compare their efficiency in detecting the genetic material of *E. multilocularis* in the samples of feces. Stool samples from red foxes were collected in a highly endemic area in Poland. Sedimentation and counting technique (SCT) was used as a reference method. (3) Results: From 48 samples, 35 were positive in SCT. Further investigations showed that 40.0% of samples (from those with SCT positive result) after Z-DNA extraction and 45.7% after Q-DNA extraction gave positive results in nested PCR. In multiplex PCR, positive results were obtained in 54.3% of samples after Z isolation and 48.6% of samples after Q. Additionally, one sample that resulted in being negative in SCT gave a positive result in PCR. The number of worms detected in the intestines had no influence on PCR results. (4) Conclusions: Both of the extraction methods showed similar efficiency in DNA isolation and dealing with inhibitors; however, they showed relatively low sensitivity. This was probably caused by degradation of genetic material in the field-collected samples.

## 1. Introduction

*Echinococcus multilocularis* is a zoonotic cestode belonging to the Taenidae family. Circulation of this parasite occurs mostly in the sylvatic cycle, based on predator–prey relationship. Typical definitive hosts for this tapeworm are canids—in Europe, mainly red foxes (*Vulpes vulpes*) but also raccoons, dogs, arctic foxes and wolves. Its intermediate hosts are small rodents, mainly from the Cricetidae family [1,2]. Humans can become an incidental intermediate host; as a consequence, this infection leads to the development of alveolar echinococcosis disease caused by larval stage of *E. multilocularis*, which is one of the most dangerous parasitic zoonosis in Europe [2]. This happens through accidental ingestion of the tapeworm’s eggs shed into the environment with feces of infected final hosts. It has been shown that domestic animals, i.e., dogs and cats, can be infected with this parasite and can become a potential source of human infections. Nevertheless, the red fox is still the crucial species responsible for contamination of the environment [2,3,4,5]. In light of the recent studies, the distribution pattern of *E. multilocularis* and prevalence in previously known areas have increased. Thus, it is important to conduct monitoring among populations of final hosts. The “gold standard” in detection of adult worms in definitive host is sedimentation and counting technique (SCT). However, since the SCT technique is applied postmortem, it is impractical for use in domestic animals. There are many alternative methods developed for recognizing *E. multilocularis* infection in definitive hosts, i.e., coproantigen detection by ELISA [6] or different PCR techniques designed for detection of the parasite’s DNA (from eggs or copro-DNA) in animal feces [7,8,9,10,11,12]. One of the advantages of the abovementioned molecular methods is their sensitivity; however, it is possible that inhibitors present in the feces may prevent the successful PCR reaction. Consequently, reported results can be false negative. In order to avoid the negative influence of inhibitors on the amplification process, there are several methods of removing them from the samples, e.g., using the inhibitor-binding substances included in commercial kits dedicated for DNA extraction from stool or using the magnetic capture (MC) technique that binds the target DNA on the magnetic beads in a very specific way [9,10].

The aim of this study was to compare two commercial DNA extraction kits combined with two PCR techniques commonly used for detection of *E. multilocularis* DNA. We evaluate their effectiveness and ability of dealing with inhibitors present in feces.

## 2. Materials and Methods 

The material for this study (48 stool samples) was collected from the large intestine of red foxes shot by hunters in the area of Podkarpackie province in Southeastern Poland. Intestines were kept in −80 °C for 7 days, for safety reasons, before examination. In a further stage, they were taken out to thaw overnight, and the next day, sedimentation and counting technique (SCT) was performed [13,14]. SCT results were treated as reference data for analyzing PCR results. During SCT protocol, stool samples were collected from posterior part of the rectum and then frozen at −20 °C, for further molecular analysis.

### 2.1. DNA Extraction 

DNA extraction was carried out for all stool samples with two extraction kits:

Z—ZR Fecal DNA Mini Prep (Zymo Research, Freiburg, Germany): An isolation was conducted in accordance with the standard manufacturer’s protocol.

Q—QIAamp FAST DNA Stool Mini Kit (Qiagen, Hilden, Germany): The isolation was performed in accordance with the special manufacturer’s protocol for DNA isolation from large volume of stool with few modifications. In the first step, 1 g of stool sample diluted in 10 mL of InhibitEX Buffer was vortexed vigorously in 50 mL centrifuge tube containing glass beads. In the first incubation, the suspension was heated at 95 °C (5 min), according to manufacturer’s recommendation for samples difficult to lyse. Second incubation was carried out at 70 °C for 20 min. In the last step, after adding ATE buffer, samples were left at room temperature for 5 min. Then, the isolates were kept in −20 °C, until further analysis.

### 2.2. Polymerase Chain Reactions (PCRs)

PCR was performed simultaneously with two different PCR methods for all DNA samples. Multiplex PCR [8] was accomplished for amplification of NADH dehydrogenase subunit 1 (nad1) of *E. multilocularis*, small subunit of ribosomal RNA (rrnS) of *Taenia* spp. and rrnS of *E. granulosus*. Nested PCR [7], with some modifications [15], was conducted for amplification of mitochondrial 12S ribosomal RNA (12S rRNA) of *E. multilocularis*. Each DNA sample was tested in undiluted (1/1) and tenfold diluted (1/10) variant. Moreover, each variant of DNA sample was tested in repetition. Internal control (DNA extracted from *E. multilocularis* adult worms) was added to one of two repeated samples, to make sure that no inhibition occurred. There was no inhibition if the specific band was present on the gel after electrophoresis.

### 2.3. Statistical Analysis

Differences in numbers of positive PCR results among two isolation methods (Z and Q) in each PCR variant were estimated by a chi-square test (or chi-square with Yates correction). The distribution of quantitative variables was tested by the Shapiro–Wilk test, and the normality hypothesis of the data was rejected. The relationship between the intensity of infection (number of *E. multilocularis* worms in the intestine assessed with SCT) and PCR results was calculated with Spearman’s rank-order correlation. The differences were considered statistically significant when *p* < 0.05. The statistical analysis was performed by using Statistica 9.1 (StatSoft Inc., Tulsa, OK, USA).

## 3. Results

SCT examination showed the presence of *E. multilocularis* tapeworms in 35 of the 48 tested samples. The intensity of infection ranged from 1 to 75,000 tapeworms per intestine.

The PCR results (for *E. multilocularis*) were compared to the results of SCT. Out of 35 samples positive for *E. multilocularis* in SCT, 23 gave a positive result, simultaneously in nested and multiplex PCR. Nested PCR showed the presence of *E. multilocularis* genetic material in 14 (after Z isolation) and 16 (after Q isolation) stool samples in total, 40.0% and 45.7%, respectively (Table 1). In multiplex PCR, 19 samples (Z) and 17 (Q) gave positive results, 54.3% and 48.6%, respectively (Table 1). There were no significant differences between Z and Q extraction methods in any variant of PCR and dilution. Twelve SCT positive samples did not give a band specific for *E. multilocularis* DNA in any of the PCRs applied, regardless of the intensity of the infection. Additionally, one negative sample in SCT, gave a positive result in multiplex PCR (only in undiluted DNA) (Q).

The analysis of relationship between number of *E. multilocularis* worms in the intestine and PCR results showed modern positive correlation for Z extraction (r_s_ = 0.5429 for nested PCR; r_s_ = 0.4629 for multiplex PCR) and low positive correlation for Q extraction (r_s_ = 0.3436 for nested PCR; r_s_ = 0.3680 for multiplex PCR).

**Figure 1:**
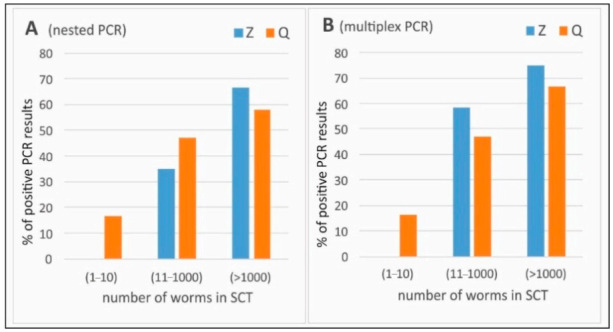
Percentages of *Echinococcus multilocularis* positive PCR results in samples of feces coming from intestines with different number of worms estimated with sedimentation and counting technique (SCT)—grouped in three levels of intensity: low (1–10 worms), medium (11–1000) and high (>1000) intensity. Z—ZR Fecal DNA Mini Prep (Zymo Research, Freiburg, Germany); Q—QIAamp FAST DNA Stool Mini Kit (Qiagen, Hilden, Germany); (A) nested PCR and (B) multiplex PCR.

Figure 1 presents percentages of positive PCR results grouped in three levels of intensity estimated with SCT: low (1–10 worms) (n = 6), medium (11–1000) (n = 17) and high (>1000) (n = 12) intensity. We observed that only the Q extraction allowed for positive PCR results in low number of worms in the intestine. Moderate- and high-intensity infection percentages of positive PCR results were similar in both extraction methods. The highest number of PCR positive results in each variant was detected in feces coming from intestines infected with a high number of worms (>1000).

Internal control gave negative results (both in diluted and undiluted DNA) only in one sample after Q isolation tested with nested PCR (in multiplex PCR it was positive). This sample after DNA extraction with the Z method gave positive results in both PCRs. Additionally, in nested PCR, undiluted samples with added internal control gave significantly more positive results after Z isolation (97.9%) than Q (83.3%) (Table 2)

*Taenia* spp. band in multiplex PCR gave a total of 42 out of 48 isolates (87.5%) after Z isolation and 91.7% after Q isolation (Table 3). There were no significant differences between method of DNA extraction in detection of *Taenia* spp. in any variant.

Product specific for *E. granulosus* was not obtained in any of tested samples by multiplex PCR.

## 4. Discussion

The present study was undertaken to compare two widely used commercial DNA extraction kits, together with two PCR protocols, i.e., multiplex PCR [8] and nested PCR [7]. We determined the efficiency of detecting *E. multilocularis* infection in final hosts by molecular detection of copro-DNA. 

Some authors reported higher sensitivity of molecular methods over widely used reference standards such as SCT or intestinal scraping technique (IST) [10,16]. Isaksson et al. [9] estimated sensitivity of magnetic capture (MC)-PCR to be 88.2%, and Dinkel et al. [7] estimated sensitivity of 89% for nested PCR. In our study, from all of the samples confirmed as positive by SCT, only 40.0–54.3% of them gave positive results in molecular tests (maximum with combination of Z extraction and multiplex PCR). A similar percentage of positive results (52%) was obtained by Maksimov et al. [17] in the stool samples from *E. multilocularis* naturally infected foxes. In Dinkel et al.’s [7] study, they showed correlation of *E. multilocularis* gravid worm number in IST and PCR sensitivity. It was on a level of 100% for samples containing 1000 and more adult tapeworms and 70% for 10 or less of immature ones. Similar dependency was observed by Maksimov et al. [17]. In their studies, PCR sensitivity was 90% for the samples taken from foxes with intense infection and only 30% for those with low infection level. In our study, we observed moderate or low positive correlation between number of worms detected in SCT and PCR results. However, it must be stressed that some of the SCT positive samples did not show positive PCR results, even though they were extracted from the stool taken from intestines of foxes with very differential number of tapeworms (from 1 to more than 1000 per sample). We obtained negative results in molecular tests both for samples with less than 10, as well as a few thousands of adult worms. Among all of the SCT negative samples, only one sample was PCR positive. In contrast, Dinkel et al. [7] used IST as a reference method. IST has been proven to be less sensitive than SCT [18,19]. Therefore, this could be a reason for the higher PCR sensitivity (compared to IST) in Dinkel’s study than in presented investigation, where SCT was used as a standard. The other factor that might have affected the detection of *E. multilocularis* DNA with PCR is that all intestine samples in this study were collected by hunters in the field. Thus, the storage and transport conditions could not be controlled in detail. With conventional PCR, Maksimov et al. [17] obtained much better sensitivity results for samples experimentally spiked with tapeworm eggs in the laboratory conditions than from naturally infected foxes. Natural conditions and DNase enzymes in the feces may influence or even damage DNA [20], resulting in a negative PCR outcome even in samples positive in SCT.

PCR amplification of genetic material extracted from stool samples can be disrupted by inhibitors present in the feces, giving false negative results. There are two most common approaches for sample treatment that can be applied in order to limit inhibitors presence and lower the risk of amplification disturbance. One of these approaches is to perform extraction not directly from stool but from tapeworm eggs separated from the sample prior to extraction [21,22]. This method is useful especially for experiments with small amounts of stool samples [22]. However, it is time-consuming, laborious and carries the risk of losing the positive material. Additionally, flotation is not so highly efficient in detecting tapeworm eggs; thus, this method may eliminate some of the potentially positive samples [23]. It is also limited because there are no eggs in the stool in early stage infections with immature worms [7]. The second approach is conducting DNA extraction from the stool and then diluting the isolate [23]. Extracting genetic material directly from feces lowers the risk of losing positive material. Diluting the DNA helps to lower the concentration of inhibitors in the final sample volume, decreasing the possibility of their influence on the amplification process. It leads not only to lower the concentration of inhibitors but also a specific DNA, so it is important to use sensitive and specific PCR protocol in order to obtain satisfactory results. Additionally, the use of an extraction method suitable for feces is a key factor. In Al-Sabi et al.’s [21] study, they obtained results with sensitivity on a very low level, by using a commercial kit dedicated for tissues. However, they isolated the genetic material from stool samples with no inhibitors eliminating step. In Karamon’s [23] study, two stool dedicated extraction kits gave significantly more positive results in comparison to the method dedicated for tissues. In order to determine if any DNA inhibition occurred, each sample was tested in repetition with internal control. In one variant (extraction kit Q combined with nested PCR), there were eight negative results of internal controls in undiluted DNA samples, while there was only one in diluted DNA. This confirms the need to examine additional diluted variants of samples, to avoid false negative results.

The impact of freezing-and-thawing process on the quality of DNA occurring in feces should also be presented. In this study, all of the intestines were frozen at −80 °C, for one week, due to safety reasons. Then, stool samples taken from the intestines during SCT preparation were frozen at −20 °C and kept for further analysis. Klein et al.’s [16] study shows that freeze–thawing cycles significantly increase PCR sensitivity, possibly by mechanical disruption of egg shells, which helps release DNA. In this method, the number of eggs prevalent in the feces influenced the result. On the other hand, repeated freezing and thawing degrades “free” DNA (DNA from tissue fragments or the whole worms shed with feces), which negatively affects the sensitivity of further molecular analysis when there are no or few parasite eggs in the sample [7,23]. False negative results may be also due the fact that, even if the eggs are present in stool, they may contain too little DNA for efficient amplification [24].

## 5. Conclusions

Our investigation showed similar effectiveness of two DNA extraction kits dedicated for stool samples in *E. multilocularis* detection. Moreover, the presented methods were found to be good at eliminating of copro-inhibitors. The study indicated the problem with relatively low sensitivity of PCRs in the examination of field samples from naturally infected animals (in comparison to microscopic examination of intestine), what was probably connected with degradation of genetic material in feces.

## Figures and Tables

**Table 1:** Percentages of PCR *Echinococcus multilocularis* positive results among fecal samples obtaining from *Echinococcus multilocularis* sedimentation and counting technique (SCT) positive intestines.

Method of Isolation	Percentage of Positive Results in *Echinococcus multilocularis*									
	Nested PCR					Multiplex PCR				
	Total	1/1	1/10	Only 1/1	Only 1/10	Total	1/1	1/10	Only 1/1	Only 1/10
Z	40.0	40.0	34.3	5.7	-	54.3	51.4	42.9	11.4	2.9
Q	45.7	45.7	25.7	20.0	-	48.6	45.7	28.6	22.9	2.9

1/1—results obtained in undiluted DNA variant; 1/10—results obtained in tenfold diluted DNA variant; only 1/1—positive results obtained only in undiluted variants (while diluted variants were negative); only 1/10—positive results obtained only in diluted variants (while undiluted variants were negative); Z—ZR Fecal DNA Mini Prep (Zymo Research, Freiburg, Germany); Q—QIAamp FAST DNA Stool Mini Kit (Qiagen, Hilden, Germany).

**Table 2:** Results in *Echinococcus multilocularis* internal controls obtained in nested PCR and multiplex PCR.

Method of Isolation	Percentage of Positive Results in *Echinococcus multilocularis* Internal Controls									
	Nested PCR					Multiplex PCR				
	Total	1/1	1/10	Only 1/1	Only 1/10	Total	1/1	1/10	Only 1/1	Only 1/10
Z	100.0	97.9 ^a^	100.0	0.0	2.1	100.0	100.0	100.0	0.0	2.1
Q	97.9	83.3 ^b^	97.9	0.0	14.6	100.0	100.0	97.9	0.0	0.0

^a.b^ Different letters in superscript indicate statistically significant differences among methods of isolation in individual variants (*p* < 0.05). 1/1—results obtained in undiluted DNA variant; 1/10—results obtained in tenfold diluted DNA variant; only 1/1—positive results obtained only in undiluted variants (while diluted variants were negative); only 1/10—positive results obtained only in diluted variants (while undiluted variants were negative); Z—ZR Fecal DNA Mini Prep (Zymo Research, Freiburg, Germany); Q—QIAamp FAST DNA Stool Mini Kit (Qiagen, Hilden, Germany).

**Table 3:** Percentages of PCR *Taenia* spp. positive results (multiplex PCR).

Method of Isolation	Percentage of Positive Results for *Taenia* spp.				
	Multiplex PCR				
	Total	1/1	1/10	only 1/1	only 1/10
Z	87.5	87.5	77.1	10.4	-
Q	91.7	89.6	64.6	27.1	2.1

1/1—results obtained in undiluted DNA variant; 1/10—results obtained in tenfold diluted DNA variant; only 1/1—positive results obtained only in undiluted variants (while diluted variants were negative); only 1/10—positive results obtained only in diluted variants (while undiluted variants were negative); Z—ZR Fecal DNA Mini Prep (Zymo Research, Freiburg, Germany); Q—QIAamp FAST DNA Stool Mini Kit (Qiagen, Hilden, Germany).
